# Case Report: Persistent delusional memories after postoperative delirium in a woman with complex cardiac surgical history

**DOI:** 10.3389/fpsyt.2025.1743329

**Published:** 2026-01-07

**Authors:** Aleksandra Stańska, Andrzej Klapkowski, Wojciech Karolak, Maciej Brzeziński

**Affiliations:** 1 Division of Quality of Life Research, Department of Psychology, Faculty of Health Sciences, Medical University of Gdańsk, Gdańsk, Poland; 2 Department of Cardiac & Vascular Surgery, Faculty of Medicine, Medical University of Gdańsk, Gdańsk, Poland

**Keywords:** cardiac surgery, case report, persistent delusional memories, postoperative delirium, valvular heart disease

## Abstract

**Introduction:**

Delirium is common after cardiac surgery and is associated with long-term cognitive and psychological morbidity. Many patients later recall vivid, often frightening delusional experiences, but sustained delusional conviction in such memories long after delirium resolution is rarely reported.

**Case description:**

We describe a woman in her late 60s with congenital aortic valve disease, three previous aortic valve operations and a recent tricuspid valve repair via right mini-thoracotomy. Her postoperative course was severely complicated by hemothorax, repeated thoracotomies, respiratory failure, sepsis, acute kidney injury, anemia and malnutrition, together with exposure to multiple centrally acting drugs (benzodiazepines, opioids, pregabalin, dexmedetomidine). While being treated on a general cardiothoracic ward she developed delirium, with disorientation, sleep-wake disruption and prominent persecutory and somatic delusions that staff were mocking her, taking humiliating photographs and “videos”, and forcing tablets into her throat. Delirium resolved with optimization of medical status, reduction of deliriogenic medications and short-term low-dose olanzapine, yet she retained a fixed belief that these ward events had truly occurred. For almost a year, including during a later admission for prosthetic valve endocarditis with paravalvular leak and an unsuccessful percutaneous closure attempt, she repeatedly reported these experiences as factual abuse despite psychotherapy and psychoeducation. Only during a subsequent elective admission, when reconsidering a high-risk redo valve operation that she ultimately declined, did she spontaneously reframe the memories as symptoms of “a mental disorder” and apologize to staff.

**Conclusion:**

This case highlights how delirium-related persecutory experiences can consolidate into persistent trauma-like autobiographical memories with prolonged delusional conviction, and underscores the need for structured delirium follow-up, explicit psychoeducation for patients and families, and close collaboration between cardiology, cardiac surgery, psychiatry and psychology.

## Introduction

Delirium is an acute neuropsychiatric syndrome with disturbances of attention, awareness and cognition and a fluctuating course, particularly common in older, medically complex and postoperative patients (1). After cardiac surgery, delirium is frequent and associated with increased mortality, prolonged hospitalization and long-term cognitive impairment (2, 4).

Beyond short-term morbidity, delirium often leaves intrusive, frightening ward or ICU memories. These frequently include hallucinations and delusional experiences and are strongly associated with later post-traumatic stress, anxiety and depression (3, 5–7). Distress may persist for months and affects both patients and relatives (8–10).

Most reports describe intrusive memories that patients recognize as at least partly unreal. Sustained delusional conviction in delirium-related experiences, persisting long after clinical resolution of delirium, is rarely documented. This case illustrates such a course in a woman with extreme somatic burden from repeated cardiac surgery, who maintained elaborate persecutory memories from a cardiothoracic ward for nearly a year, before ultimately reinterpreting them as illness-related phenomena.

## Patient information

The patient is a woman in her late 60s with congenital aortic valve disease and major cardiovascular history of:

- aortic valvuloplasty in childhood (1965),
- mechanical aortic valve replacement (1979),
- redo mechanical aortic valve replacement (1997, Medtronic Hall 23),
- long-standing ascending aortic aneurysm involving the brachiocephalic trunk,
- complete atrioventricular block with pacemaker implantation (VVI, later DDD) and several lead revisions,
- paroxysmal, later persistent atrial fibrillation, chronic heart failure, chronic kidney disease, hypertension and dyslipidemia.

A transient ischemic attack in 2015 left mild left-sided pyramidal signs. Cranial CT then showed no clearly demarcated ischemic lesion; a 6-mm hyperdense focus in the right cerebral peduncle was interpreted as artefact or benign deposits. Follow-up CT in June 2023 revealed only mild age-appropriate cerebral atrophy without focal ischemia or hemorrhage.

Psychiatric history included long-standing mixed anxiety-depressive disorder, with previous psychiatric hospitalization but no documented psychosis. She had been under outpatient psychiatric care and taking paroxetine for several years. She did not misuse substances, lived with her husband, had strong family support and was independent in activities of daily living.

## Clinical findings and index surgical course

In late 2023 she was admitted with progressive exertional dyspnea (NYHA IV), peripheral edema and atypical chest discomfort. Echocardiography showed severe tricuspid regurgitation with right ventricular dysfunction, preserved left ventricular function and a normally functioning mechanical aortic valve. After multidisciplinary discussion she was referred for tricuspid valve repair. On 8 December 2023 she underwent right mini-thoracotomy with tricuspid annuloplasty using a rigid ring under cardiopulmonary bypass (redo procedure).

The postoperative course was complicated by a large right hemothorax requiring drainage, recurrent pleural collections and two further thoracotomies (January and February 2024) with evacuation of ~1700 ml blood and clots and partial right lung decortication. She required prolonged ICU stay with mechanical ventilation followed by high-flow nasal oxygen, vasoactive support, broad-spectrum antibiotics and antifungals, and nutritional support.

Inflammatory markers were markedly increased (C-reactive protein repeatedly >150 mg/L, peaking above 230 mg/L). Serum creatinine rose from chronic kidney disease baseline to around 1.3–1.6 mg/dL, with urea up to ~160 mg/dL. Hemoglobin fell to 8.7–9.4 g/dL despite transfusions. Despite acute kidney injury, urine output remained adequate and renal replacement therapy was not required during the ICU stay.

After initial stabilization in ICU, she was transferred to a general cardiothoracic ward, where most subsequent complications were managed and where all prominent psychotic symptoms emerged. Throughout, she received regular psychological support and initially remained fully oriented, with reactive low mood and anxiety but no psychosis. The main cardiac, neurological and psychiatric events are summarized in **Table 1**.

**Table 1:** Timeline of cardiac, neurological and psychiatric events.

Period/date	Cardiac/somatic events	Neurological/psychiatric events
1965–1997	Aortic valvuloplasty; mechanical AVR; redo AVR; later pacemaker	Post-op transient left-sided weakness; no documented psychosis
2015–2023	TIA; CT head with mild atrophy only; progression of valve disease	Mixed anxiety-depressive disorder under outpatient care
Dec 2023	Tricuspid valve repair under CPB; ICU stay, ventilation, vasopressors	Pre-op assessment: anxious, non-psychotic
Dec 2023–mid Jan 2024	Hemothorax; repeated thoracotomies; AKI; sepsis; malnutrition	Psychological support; initially oriented
Late Jan–early Feb 2024	Treatment on general ward	Delirium with disorientation and persecutory/somatic delusions
Mid Feb 2024	Somatic improvement; discharge planning	Delirium resolves; **persistent delusional memories**
2024–early 2025	Infective endocarditis; paravalvular leak; failed percutaneous closure	Psychotherapy; fixed belief in ward persecution
Late 2025	Reassessment for high-risk redo surgery; patient declines operation	Gradual insight; memories reframed as illness-related

## Timeline

## Diagnostic assessment

### Delirium episode

In late January 2024, shortly after thoracic re-operations, she developed fluctuating confusion while on a multi-bed cardiothoracic ward. Notes describe disorientation (including stating the year as 1924), impaired concentration and memory, severe sleep-wake disruption, psychomotor agitation with attempts to leave bed (“*I am going to the city*”), repeated removal of oxygen devices and mood lability with anxiety and tearfulness.

Thought content became dominated by persecutory and referential ideas: beliefs that staff were mocking and humiliating her; that nurses were taking photographs and showing “films” of her and her husband on the ward; that tablets were being “poured into her throat” or forced against her will; and that staff, including the psychologist, were plotting to “destroy” or “degrade” her. She at times insisted that her husband was dead (untrue). Symptoms worsened in the evenings.

Psychiatric examination showed clouded consciousness with fluctuating inattention and partial disorientation to time but preserved self-identity and awareness of being in hospital. There were no clear hallucinations on examination, though descriptions suggested possible nocturnal misperceptions. Neurological status was unchanged from baseline.

Laboratory abnormalities (high CRP, anemia, renal impairment) were consistent with the complicated postoperative state. Prior CT scans had shown no focal structural lesions. Because the clinical picture fully matched delirium in the context of severe medical illness, further imaging or EEG was not pursued.

The working diagnosis was delirium due to multiple postoperative complications and systemic illness, consistent with DSM-5 criteria (1). Primary psychosis or mood disorder with psychotic features was considered unlikely given the acute onset, fluctuating course and temporal association with somatic instability.

### Post-delirium phase

Over the following days, attention and orientation normalized. By mid-February she was fully oriented, able to discuss her medical situation coherently and cognitively intact on bedside assessment. However, she continued to describe the ward experiences as entirely real. She recounted them in detail to staff, family and later outpatient therapists as factual events, not as dreams or doubts.

During later psychiatric assessments, including a 2024 admission for infective endocarditis, she displayed no new psychotic symptoms, had preserved insight in all other domains and understood her medical condition and treatment. These features supported an interpretation of persistent delusional memories linked to delirium, rather than a primary chronic delusional disorder (3, 5–7).

## Therapeutic intervention

### During delirium

Management followed multimodal delirium care principles (1, 2, 8): optimization of oxygenation, hemodynamics, infection control and renal function; reduction of deliriogenic drugs where feasible; frequent reorientation, mobilization and sleep promotion; and family involvement. Melatonin was not used as part of the sleep promotion strategy in this case.

In ICU she received dexmedetomidine infusion for several days during the most unstable respiratory period. Around the delirious episode she had also been exposed to several centrally acting drugs with known deliriogenic potential: alprazolam (0.25 mg three times daily, then tapered and stopped by 1 February), diazepam on several days, pregabalin 75 mg twice daily, a single dose of hydroxyzine, repeated oxycodone for postoperative pain and isolated doses of tramadol and morphine.

Because of agitation, distress and risk behaviors, low-dose olanzapine 5 mg orally once daily was introduced. Paroxetine was continued and later titrated for anxiety-depressive symptoms.

### After discharge

After discharge she continued outpatient psychiatric follow-up and engaged in psychotherapy, largely to cope with “what had happened” to her during the previous hospitalization, which she remained convinced was real. Therapy focused on processing the traumatic surgical course and ward experiences, exploring mistrust and anger towards staff, and managing health-related anxiety and depressive reactivity.

Despite this, for many months she firmly maintained that staff had taken humiliating photographs and “films” and had forced tablets into her throat. Anger and mistrust were often directed at the ward psychologist, whom she felt had collaborated with the nurses. Psychoeducation about delirium and contrary information from staff and family did not initially reduce her conviction.

No maintenance antipsychotic was prescribed because there were no new psychotic symptoms and potential metabolic and cardiac adverse effects were considered disproportionate to the expected benefit.

## Follow-up and outcomes

### Medical course

Within the following year she developed prosthetic valve infective endocarditis due to *Staphylococcus epidermidis* with paravalvular leak around the mechanical aortic valve, requiring prolonged intravenous antibiotics. A percutaneous attempt to close the leak in early 2025 was unsuccessful.

In late 2025 she was re-assessed for high-risk redo valve surgery. After multidisciplinary discussion she ultimately declined further operative treatment and opted for conservative management, reporting that she preferred to adapt to functional limitations rather than face another major operation.

Despite advanced heart disease, she remained cognitively functional and engaged in care. Throughout this period, she repeatedly described the earlier cardiothoracic ward stay as a time of humiliation, mockery and covert filming by staff.

### Psychological course and eventual insight

During a 2024 admission for infective endocarditis, she again requested psychological support for low mood and fear of further surgery. Her cognitive status was normal and no new psychotic symptoms were observed, yet she remained certain that her previous ward experiences had been real. Supportive counselling and psychoeducation were provided, but her beliefs did not immediately change.

During a subsequent elective cardiac surgery admission in November 2025, she was seen again by the same psychologist. She stated that, over the preceding months, she had been reconsidering these experiences. The memories remained vivid but were now described as “dream-like”. She reported that she had come to believe they were probably due to “a mental disorder” or brain disturbance after the operation, explicitly denied that staff had truly mocked or filmed her, apologized for her earlier accusations and expressed relief that she could clarify the situation and “not leave a bad impression”.

## Discussion

### Somatic and perioperative risk factors

This case illustrates cumulative risk factors for postoperative delirium and later neuropsychiatric sequelae: older age; congenital and acquired heart disease; chronic kidney disease and hypertension; three previous cardiac operations under cardiopulmonary bypass; a fourth high-risk redo procedure; hemothorax, repeated thoracotomies, anemia, systemic inflammation and metabolic disturbance; prolonged ICU stay followed by complex ward care; and exposure to benzodiazepines, opioids and gabapentinoids (1, 2, 4, 8). Cerebrovascular vulnerability after TIA and pre-existing anxiety-depressive disorder further increased susceptibility to delirium and PTSD-like reactions (3, 5–7).

### Persistent delusional memories as a delirium sequela

Delusional ward or ICU memories with persecutory themes are common and strongly associated with subsequent PTSD and depression (3, 5–7). In most descriptions, however, patients retain some sense that these experiences were not fully real.

In this patient, conglomerated ward memories functioned phenomenologically as fixed delusional beliefs for almost a year, despite intact cognition, absence of new psychotic phenomena and ongoing psychotherapy. Only with time, repeated information and a further stabilization of her medical status did she reframe them as illness-related. This supports the notion that delirium-related memories can consolidate into trauma-like autobiographical episodes with sustained delusional conviction, blurring the boundary between psychosis and memory disturbance.

The systematic review by Danielis et al. highlights how patients struggle to place such experiences on a real – unreal continuum and may need time and structured support to integrate them into a coherent narrative (7). Our case extends these findings by showing that this integration can be markedly delayed and that insight may emerge only during later admissions, when emotional and somatic context has changed.

Her eventual refusal of further valve surgery can also be understood psychosomatically as an attempt to restore agency over her body after decades of invasive interventions and a particularly traumatic postoperative course.

### Psychotherapy, timing and relational impact

The limited early impact of psychotherapy likely reflected subtle cognitive deficits after delirium, strong emotional “tagging” of experiences during extreme physiological stress and the absence of a structured reconstruction of events that might have helped anchor her memories (3–7, 9). The turning point occurred only when the therapeutic relationship could be revisited in a more stable phase.

Family members were distressed by her allegations of abuse yet trusted the medical information, creating loyalty conflicts. Staff, including the psychologist, experienced moral distress at being perceived as perpetrators. Early, honest communication about delirium and the possibility of false but convincing memories may help reduce such relational damage and facilitate later re-appraisal (8–10).

### Strengths and limitations

Strengths include detailed longitudinal data across multiple admissions, integration of somatic and psychiatric perspectives and inclusion of the patient’s later reflections. Limitations are the single-case design, absence of standardized delirium ratings and lack of neuroimaging or EEG at the time of delirium, precluding comment on acute structural or electrophysiological changes.

## Conclusions

This case demonstrates that:

- Severe postoperative delirium after complex cardiac surgery, evolving on a general cardiothoracic ward, can lead to persistent, vividly remembered persecutory experiences that behave like fixed delusional beliefs.
- Such delirium-related memories may remain resistant to early psychoeducation and psychotherapy, even in patients with good premorbid functioning and continuing psychiatric care.
- Insight and re-appraisal may emerge slowly and non-linearly, facilitated by time, further therapeutic contact and integration of medical and psychological narratives.
- Families and staff require support in coping with the emotional impact of persistent delusional memories and allegations of abuse.

Explicitly naming this phenomenon may help clinicians normalize it for patients and relatives and design follow-up pathways that address not only cognitive and functional outcomes but also the subjective consequences of delirium.

## Patient perspective

The patient described the delirious period as “terrifying” and “humiliating”, with a firm belief that staff were laughing at her, taking pictures and showing films of her and her husband. For many months she felt angry and betrayed, particularly by the psychologist whom she believed had sided with the nurses.

Over time, discussions with her family, psychiatrist and cardiology team led her to reconsider these experiences. She now believes they were symptoms of “a mental disorder” related to the severity of her illness and the medications she received. The memories remain vivid but are now “like a dream” rather than proof of real persecution. She expressed relief at being able to meet the psychologist again and apologized for her previous accusations.

She also reflected on her decision not to undergo another valve operation, stating that she was aware of the high perioperative risk and preferred to adapt to her current limitations rather than face another major surgery.

## Data Availability

The data analyzed in this study is subject to the following licenses/restrictions: The dataset consists of identifiable clinical records from a single patient and cannot be shared publicly due to confidentiality and privacy regulations. Only de-identified, clinically relevant information is reported in the article. Additional anonymized details could be made available from the corresponding author on reasonable request, subject to institutional policies and patient consent. Requests to access these datasets should be directed to Aleksandra Sta&nacute;ska, astanska@gumed.edu.pl.
